# Roles of FGF signaling in stem cell self-renewal, senescence and aging

**DOI:** 10.18632/aging.100369

**Published:** 2011-10-09

**Authors:** Daniel L. Coutu, Jacques Galipeau

**Affiliations:** ^1^ Stem Cell Dynamics Research Unit, Helmholtz Zentrum München, 85764 Munich (Germany); ^2^ Departments of Hematology & Medical Oncology, and Pediatrics, Emory University, Winship Cancer Institute, Atlanta 30322, GA (USA)

**Keywords:** fibroblast growth factor, self-renewal, senescence, aging, adult stem cells, embryonic stem cells

## Abstract

The aging process decreases tissue function and regenerative capacity, which has been associated with cellular senescence and a decline in adult or somatic stem cell numbers and self-renewal within multiple tissues. The potential therapeutic application of stem cells to reduce the burden of aging and stimulate tissue regeneration after trauma is very promising. Much research is currently ongoing to identify the factors and molecular mediators of stem cell self-renewal to reach these goals. Over the last two decades, fibroblast growth factors (FGFs) and their receptors (FGFRs) have stood up as major players in both embryonic development and tissue repair. Moreover, many studies point to somatic stem cells as major targets of FGF signaling in both tissue homeostasis and repair. FGFs appear to promote self-renewing proliferation and inhibit cellular senescence in nearly all tissues tested to date. Here we review the role of FGFs and FGFRs in stem cell self-renewal, cellular senescence, and aging.

## INTRODUCTION

In the 1960's, Hayflick observed that human cells displayed a finite lifespan when cultured in vitro [1]. He later determined that most cells had a maximal capacity to proliferate in vitro of about 50 population doublings (the Hayflick limit) after which they entered what he termed cellular senescence, a process characterized by irreversible growth arrest [2]. These observations led him to propose a cellular theory of aging whereby cellular senescence accounts for the aging process and on the contrary, escape from senescence leads to cellular transformation and cancer. This theory is still widely accepted today although direct proof of it is lacking. It is also still debated whether cellular senescence causes aging or conversely if aging causes cellular senescence [3, 4]. Nevertheless, there is an increasing amount of experimental data demonstrating an accumulation of senescent cells in aged tissues [3, 5].

Cellular senescence can be caused by intrinsic or extrinsic factors and this distinction is important [6]. Intrinsic senescence is caused by telomere shortening, which occurs after each cell division. Cells that do not express telomerase thus have a limited number of possible cell divisions before genomic instability ensues. This triggers the p53, p21 and pRb pathways to promote growth arrest and cellular senescence. Because murine cells have very long telomeres, they are not believed to undergo intrinsic senescence in normal conditions. Indeed, mice lacking telomerase activity only show signs of accelerated aging after six generations [6]. However, murine cells are also renowned for their high rate of transformation when cultured in vitro. This usually occurs after very few population doublings when the cells enter a crisis phase and stop proliferating. Although most of those cells do not survive, some transformed and immortalized clones often arise from the culture and display a high degree of genomic instability and a propensity for tumorigenesis. This type senescence that precedes transformation is thought to be caused by artificial laboratory culture conditions (such as high oxygen) and is referred to as extrinsic senescence. It mainly involves the p16^INK4a^ pathway in human cells and also the p19/ARF pathway in murine cells. In human cells, both intrinsic and extrinsic senescence can thus coalesce to play a role in aging.

**Figure 1:**
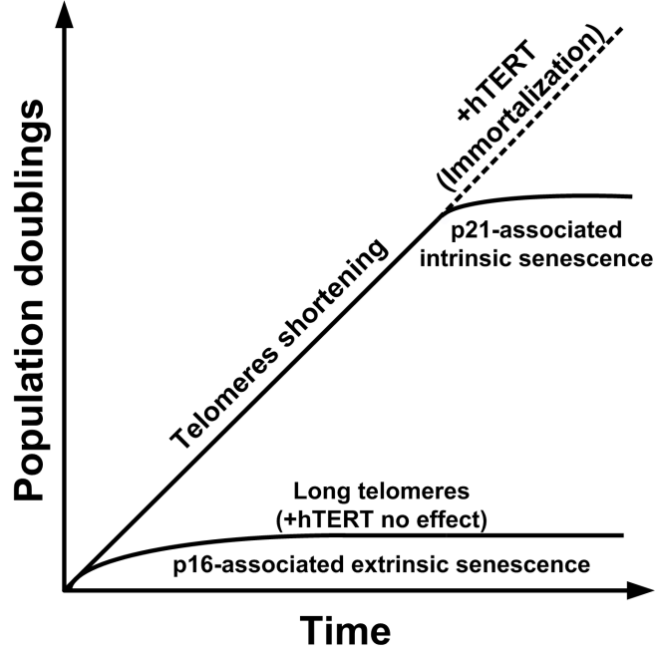
Mechanisms of cellular senescence in human cells. In normal proliferating human cells, telomeres at the end of chromosomes are shortened at every cell division unless the cells express telomerase. When telomeres get too short, genomic instability ensues and a DNA-damage response under the control of the p21 pathway is induced. This causes growth arrest and intrinsic cellular senescence. Transduction of these senescence cells with a telomerase construct reverses this growth arrest and leads to immortalization. When cells undergo stress (e.g. reactive oxygen species, ionizing radiations, etc.) they can undergo p16-mediated extrinsic senescence even though they possess long telomeres. Re-expression of telomerase in this case does not rescue this irreversible growth arrest. Murine cells have very long telomeres and are not thought to be susceptible to intrinsic senescence in normal conditions. However, they are very sensitive to extrinsic senescence. Murine cells often escape from p16-mediated senescence and get immortalized but the mechanism for this is unclear.

The process of aging is a systemic degenerative process caused by intrinsic (genetic, epigenetic) and extrinsic (environmental) factors. It affects multiple organs, mainly those with a high metabolic demand or those which are mitotically active and require constant or frequent regeneration [7]. As such, aging is associated with a decrease in the regenerative properties of many tissues including bone, skin, muscle, brain and more. Adult or somatic stem cells have been identified in almost every organ tested: skin, intestine, bone and bone marrow, liver, heart, brain, pancreas, etc. These stem cells are thought to sustain tissue growth, homeostasis and repair throughout the lifetime of the organism. In consequence, the blunted regenerative potential of tissues observed during aging may be viewed as a stem cell disorder, where stem cells are lost or inactivated by senescence.

Adult stem cells provide constant replacement cells for tissue homeostasis and repair while at the same time maintaining a pool of stem cells by the process of self-renewal, where following cell division at least one daughter cell is still a stem cell whereas the other is either a stem cell (symmetric division) or a differentiated progeny (asymmetric division). The stem cell pool only regresses if a symmetric division giving rise to two differentiated progeny occurs, or if the stem cell undergoes cellular senescence (these two processes not being exclusive). In recent years, a number of studies have identified fibroblast growth factors (FGFs) and their receptors (FGFRs) as key regulators of both senescence and self-renewal in a variety of stem cell types.

FGFs (23 known members) and FGFRs (5 known members, expressed as multiple splice variants) have long been known for their important roles in embryonic development [8, 9]. However, the vast number of somewhat redundant ligands and receptor variants, as well as the promiscuous ligand usage by the receptors has made it difficult to study the roles of FGFs/FGFRs using genetic methods [10]. Furthermore, FGF signaling is modulated by tissue specific heparan-sulfate proteoglycans (HSPGs) that either inhibit or amplify FGFR activation. The divergent effects of FGF signaling also appear to depend on the state of differentiation of the cells, the repertoire of FGFRs they express, and the presence of other growth factors or cytokines. Nevertheless, as further tools and reagents are developed, a more comprehensive image is starting to emerge.

The purpose of this Research Perspective article is to review the roles of FGFs and FGFRs in different stem cell populations and highlight their roles in stem cell self-renewal, cellular senescence and aging.

## FGF SIGNALING IN EMBRYONIC DEVELOPMENT AND EMBRYONIC STEM CELLS

In early murine embryonic development FGF-4 is the first member to be expressed, from the 4 cell stage onto the blastocyst, egg cylinder and primitive streak [11]. Its deletion causes peri-implantation embryonic lethality (E4-5); early development appears normal up to the blastocyst stage but embryos die within hours after implantation owing to deficient inner cell mass formation and maintenance [12]. FGF-4 signaling appears to be important as early as the fifth cell division to promote cell proliferation onto the blastocyst stage [13]. FGF-4 probably signals through FGFR2 as this receptor is the first detected in development, although early expression of FGFR1, 3 and 4 have also been inconsistently reported (probably owing to the few reliable antibodies available)[14, 15]. Moreover, FGFR2 deletion recapitulates FGF-4 deletion, causing early embryonic lethality (E6-8) due to defects in inner cell mass. FGFR1 deletion is also lethal (E7.5-9.5) and appears to cause defects in gastrulation, mainly by affecting axial patterning and migration/proliferation of cells through the primitive streak, thus inhibiting mesoderm and endoderm specification [16, 17]. FGFR3 deletion on the other hand is not embryonic lethal but mice display skeletal malformations that may lead to premature death (see section on skeletal/mesenchymal stem cells below), whereas FGFR4 null mice show no obvious phenotype. The functions of this latter receptor in development and postnatal life remain unclear as well as that of FGFR5.

**Figure 2:**
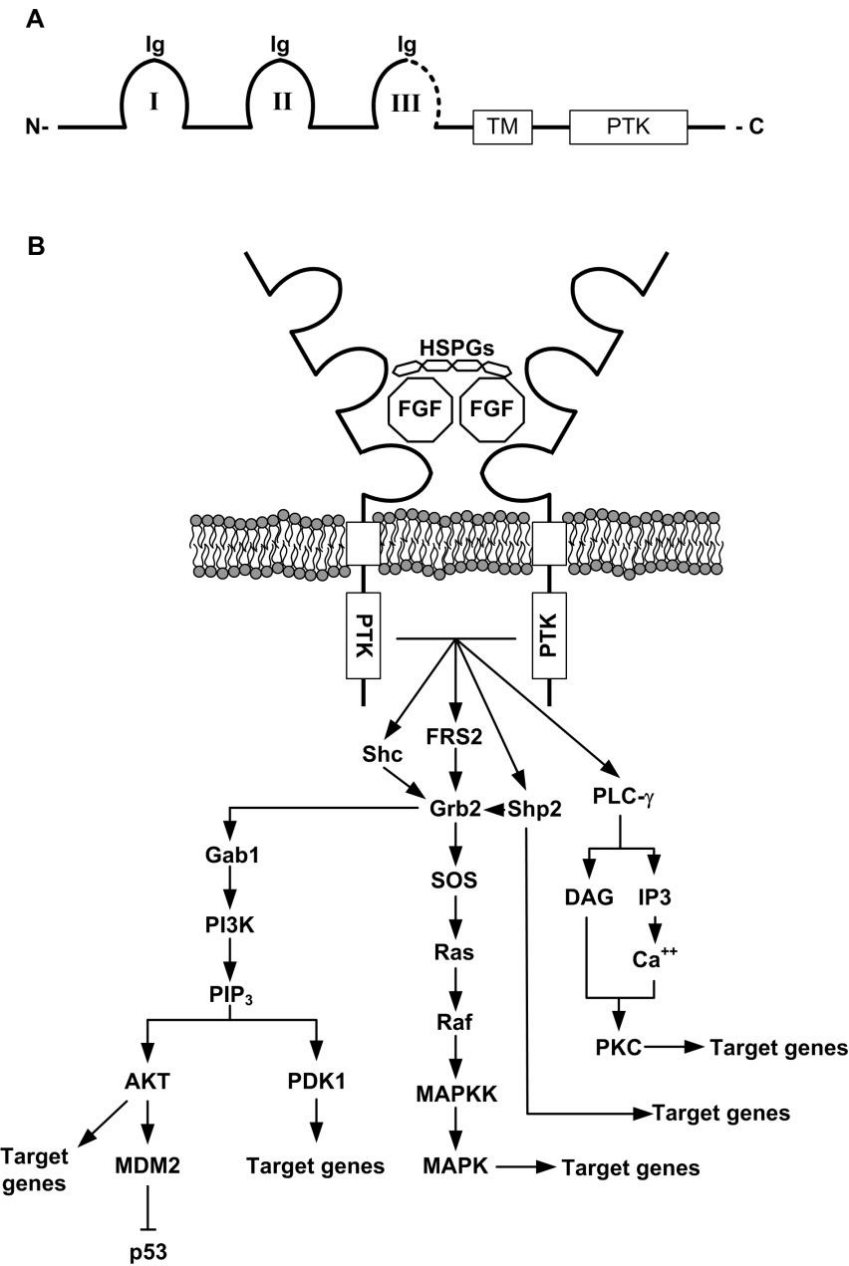
Structure and signaling downstream of FGFRs. A) FGFRs possess three extracellular immunoglobulin-like domains (Ig I to III), a transmembrane domain (TM) and an intracellular protein tyrosine kinase domain (PTK).The third Ig-like domain (III) is thought to confer ligand specificity. The C-terminal half of this IgIII domain (dotted line) is alternatively encoded by either exon 8 or 9 of the receptor gene, which create the two main isoforms of FGFR1, 2 and 3 (IIIb for exon 8 and IIIc for exon 9). Other isoforms also exist (no PTK domain, no TM domain) but are less abundant. B) Creation of the ternary complex between heparan sulfate proteoglycans (HSPGs), FGF ligands, and FGFRs leads to autophosphorylation of the PTK domains and activation of a number of intracellular pathways downstream. FRS2 and Grb2 and the main mediators of the signaling and activate various effectors such as PI3K/AKT and MAPKs. Other pathways (Shp2, PLC-γ) are also activated. Note that activation of the PI3K pathway can lead to phosphorylation of MDM2 on Ser186, leading to its translocation to the nucleus and subsequent degradation of p53.

Murine embryonic stem cells (mESCs) have historically been derived from the inner cell mass of the blastocyst [18, 19] or the epiblast (although these ESCs are considered more primed for gastrulation and germ layer commitment). Since FGF-4^−/−^ embryos fail to develop because of defects in inner cell mass proliferation and germ layers specification, it has been assumed that FGF signaling in mESCs was required for their differentiation or lineage commitment. Of note, mESCs constitutively express FGF-4 which is thought to act in an autocrine manner. Undifferentiated mESCs were found to express high levels of FGFR1 and 4 which are maintained during differentiation [15]. They also express FGFR2(IIIb) and FGFR3(IIIc) but upregulate FGFR2(IIIc) and FGFR3(IIIb) upon differentiation. FGF-4^−/−^ mESCs do not display defects in proliferation in vitro and are capable of multilineage differentiation, however the survival of those differentiated progeny is severely compromised, although the underlying mechanism for this phenotype is still unclear [20]. Further studies using FGF-4^−/−^ mESCs or specific inhibitors of FGFR1 and 3 confirmed that inhibition of FGF signaling through these receptors could maintain mESCs in a self-renewing, pluripotent state [21, 22]. However, these studies also suggest that FGFR1/3 signaling, as well as the presence of specific HSPGs, may act more as a priming or permissive signal for differentiation rather than a differentiation cue itself. Taken together, these studies suggest that FGF signaling in murine embryogenesis and ESCs may have stage specific effects. FGF-4 signaling through FGFR2 stimulates ESCs proliferation from the fifth division to the establishment of the inner cell mass in the blastocyst, whereas signaling through FGFR1/3 in peri-implantation embryos and epiblast ESCs is important for germ layer specification. However, the exact timing of expression of the various receptor isoforms in early lineage specification and their role in self-renewal, priming and differentiation of mESCs remains unclear to date.

The study of molecular events in human post-implantation embryogenesis is complicated by ethical and technical limitations. It is however possible to study pre-implantation embryos from which are derived humans ESCs. It must be noted that hESCs might be more related to epiblast-derived (primed) mESCs in terms of properties and functionality. Nevertheless, hESCs have been found to express several molecular-mass isoforms of FGF-2, 11 and 13 (but not FGF-4) as well as the whole repertoire of FGFRs with the following relative abundance (mRNA levels): FGFR1 > FGFR3 > FGFR4 > FGFR2 [23]. These levels are modulated during hESCs differentiation, showing an initial decrease followed by upregulation in more advanced differentiation. Early evidence suggested that FGF signaling might be important for proliferation and self-renewal of hESCs in vitro [24-27]. These observations were confirmed by many groups and to date FGF-2 is a necessary supplement to hMSCs culture medium, independently of the presence or absence of a feeder layer. The maintenance of pluripotency (self-renewal) by FGF-2 on hESCs may be in part attributed to modulation of Wnt signaling through PI3K [28], but a recent study again suggests that its effects may be stage specific or dependent on context [29]. Indeed, FGF-2 may also be important to sustain Nanog expression during BMP-4 induced differentiation of hESCs and promote mesendoderm over trophoectoderm differentiation.

In summary, the roles of FGF signaling in murine and human embryogenesis are diverse and appear stage specific. They are probably modulated by the context (presence and type of HSPGs), the differentiation status of target cells, and the repertoire of FGFRs these cells express. However, FGF signaling is important for self-renewal and proliferation of primitive ESCs and for migration, proliferation and lineage commitment of more differentiated cells.

## FGF SIGNALING IN MESODERMAL STEM CELLS AND TISSUES

“Fibroblast growth factor” was first isolated in 1974 from bovine pituitary gland and shown to have a mitogenic effect on many cells types [30]. The prototypical FGF ligands, acidic FGF (aFGF or FGF-1) and basic FGF (bFGF or FGF-2), were then purified by heparin affinity chromatography in the early 1980's as the first potent endothelial cells mitogens [31-33]. Latter observations showed that FGF signaling inhibition impairs mesodermal patterning and bone formation, and that mutations in FGFRs cause skeletal abnormalities in mice and humans. It is thus not surprising that the FGFs/FGFRs systems have been mostly studied in mesodermal and mesenchymal tissues to date. This section will review the roles of FGF signaling in three mesoderm-derived tissues and their associated stem cells: skeletal tissue, vascular tissue and hematopoietic tissue.

### Mesenchymal stem cells and skeletal tissues

The importance of FGF signaling in skeletal tissues was first highlighted by genetic linkage analysis demonstrating that the etiology of achondrodysplasia (one of the most common forms of dwarfism in humans) was due to activating point mutations in FGFR3 [34, 35]. Other forms of skeletal dysplasias were then linked to FGFR1 and 2 mutations. Broadly, these conditions can be divided in two categories: achondrodysplasias and craniosynostosis syndromes. Some particularly severe achondrodysplasias are postnatally lethal within a few months. They are usually caused by activating mutations in FGFR3, which suggests that this receptor is a negative regulator of chondrogenesis. The craniosynostosis syndromes are characterized by premature cranial sutures fusion but are also associated with appendicular skeletal malformations and mental retardation. Most of them are associated with activating or gain-of-function mutation in FGFR2 but others link to FGFR1 and 3. These mutations lead to increased osteoblast differentiation and maturation, implying a role for these receptors as positive regulators of osteogenesis.

In the early stages of bone development, FGFR1(IIIc) is expressed in limb mesenchyme whereas FGFR2(IIIb) is expressed in overlying ectoderm [36]. There appears to be an intimate crosstalk between these tissues as mesenchyme-derived FGF-10 signals through ectodermal FGFR2b to initiate apical ectodermal ridge (AER) formation and induces FGF8 expression, which in turn activates mesenchymal FGFR1c. At the condensation stage, FGFR1 continues to be expressed in loose mesenchyme and in the condensation whereas FGFR2 is expressed solely in the condensation [37]. At later stages of development, FGFR1 and 2 are still expressed in perichondrium and periosteum and FGFR1 can also be observed in osteogenic lineage cells within the marrow cavity, endosteum and trabecular bone [34]. FGFR3 is expressed by proliferating chondrocytes at the onset of chondrogenesis and is maintained until growth plate closure. When chondrocytes stop proliferating to become prehypertrophic, they down regulate FGFR3 and upregulate FGFR1. Perichondrium-derived FGF18 appears to activate FGFR3 on proliferating chondrocyte to limit their proliferation [38-40].

Because FGFR1 and 2 knockout murine embryos die before skeletal development, conditional knockout techniques have been used to elucidate the roles of these receptors in bone lineage cells. FGFR1 signaling in osteogenic cells appears to have developmental stage-specific effects. When inactivated at the early condensation phase in brachyury expressing cells, a decreased proliferation of mesenchymal progenitors is observed, along with decreased condensation sizes and numbers and delayed patterning (segmentation, branching)[41]. When inactivated in collagen 2 expressing osteo-chondro-progenitors, osteoblasts showed delayed maturation based on collagen 1 and osteopontin expression, but normal commitment to the osteoblast lineage based on Runx2 mRNA expression [42]. When FGFR1 is inactivated in mature, collagen 1 expressing osteoblasts, the resulting phenotype suggest an accelerated differentiation resulting in increased trabecular volume and mineralization [42]. FGFR1 thus seems to be required at different stages of bone cell development: 1) it stimulates limb bud elongation in the proximal-distal axis; 2) it increases mesenchymal progenitors proliferation and survival; 3) it is involved in the patterning of the skeletal elements; 4) it is required for commitment of the progenitors to the osteoblast lineage; and 5) it inhibits terminal differentiation of osteoblasts.

A similar strategy has been used to study the role of FGFR2 in bone lineage cells. Conditional inactivation of this receptor in Dermo1 (Twist2, expressed in the mesenchymal condensation giving rise to both osteoblasts and chondrocytes) expressing cells resulted in severe dwarfism accompanied by reduced bone mineral density [43]. At E16.5, normal levels and distribution of Runx2, osteopontin, collagen 1 and osteocalcin were observed but were drastically reduced in postnatal animals, reflecting a decreased osteoblast number. Significantly less trabecular bone was formed in conditional knockout animals and trabecular osteoblasts appeared atrophic and disorganized. Perichondrium and periosteum also showed decreased thickness, with reduced osteoblasts number and mineral apposition rate. Osteoblasts and progenitors proliferation was reduced in perichondrium, trabecular bone and cortical bone. In another study, the mesenchymal isoform (IIIc) of FGFR2 was disrupted by inserting a stop codon in exon 9 of the FGFR2 gene [44]. These mice also exhibit dwarfism with skeletal defects in both cranial and long bones. These mice have a delayed onset of mineralization, early synostosis caused by a loss of proliferating osteoblasts, deficient growth of the skull base and a narrowing of the hypertrophic chondrocyte layer in the growth plate of long bones. The different phenotypes observed in these two mouse models could be explained by possible alternative exon usage in the latter, although the expression of FGFR2(IIIb) was reported normal at least between E12.5 and E14.5. Nevertheless, we can conclude from these studies that FGFR2 is an important regulator of osteoprogenitors proliferation and of the anabolic function of mature osteoblasts.

The role of FGF signaling in skeletal cells has also been studied in vitro in mesenchymal stem cells (skeletal stem cells), which are thought to give rise to all skeletal or mesenchymal cells in bones and sustain bone homeostasis and repair throughout life. The mitogenic effect of FGF on mesenchymal stem cells (MSCs) was first described over 20 years ago [45]. Several subsequent studies have confirmed this observation and showed that FGF signaling maintains MSCs in an undifferentiated state during proliferation while preserving their multipotentiality [46-53]. In other words, FGF appears to promote self-renewal and maintain stemness of MSCs in vitro. However, the molecular mechanisms underlying this effect have only recently been investigated in more details. Mansukhani et al. (2005) provided evidence that signaling through FGFR2 inhibits osteoblast differentiation by inducing the expression of the pluripotency marker Sox2, which antagonizes Wnt signaling (a postitive regulator of bone formation) by binding to and inhibiting β-catenin [54]. The same group later demonstrated that Sox2 was required for self-renewal of osteogenic cells [55]. On the other hand, FGF signaling has also been described as a negative regulator of MSCs senescence in both human and mouse [56-59]. More specifically, we have shown that MSCs express both FGFR1 and 2 and that FGF stimulation is absolutely required to avoid extrinsic senescence of murine MSCs [58]. FGFR signaling in MSCs induces phosphorylation of MDM2 on serine 186 in a PI3K/AKT-dependent manner. This post-translational modification releases MDM2 from its inhibitor p19/ARF, induces its nuclear translocation and enhances its ubiquitin-ligase activity as well as its affinity for p53, targeting the latter for proteosomal degration [60, 61].

The observations that FGF acts as a mitogen (probably by ERK1/2 activation), a multipotency factor (through Sox2 induction) and an inhibitor of cellular senescence (through a PI3K-AKT-MDM2 pathway) are significant in that they may explain how MSCs are capable of maintaining a sufficient pool of progenitors during bone development, growth, homeostasis and repair for the lifespan of the organism. These observations also provide potential therapeutic targets as senescence of osteoblasts and their progenitors is an important cause of age-associated bone loss and osteoporosis [62]. Data from both conditional knockout experiments and in vitro experiments using MSCs suggest that FGF signaling may act as a balancing factor to maintain the size of the skeletal progenitor pool while avoiding overgrowth, by stimulating stem cell proliferation while inducing committed progenitor differentiation.

### Endothelial progenitor cells and vasculature

In embryonic development, mesodermal cells arising from the posterior primitive streak are thought to migrate to the yolk sac and to the para-aortic splanchnopleura (precursor to the aorta-gonad-mesonephros, AGM) and differentiate into haemangioblasts, common progenitors of both endothelial and hematopoietic cells [63, 64]. As already mentioned, FGF-2 was the first potent angiogenic factor identified in the early 1980's, which appears to signal exclusively through FGFR1 in endothelial cells (ECs). However, the specific role of FGF signaling in ECs remains elusive. It has proven difficult to study, largely because: 1) FGFR1 knockout mice die shortly after gastrulation and before the onset of vascularisation, 2) ECs are highly heterogeneous in terms of markers expression and phenotype, precluding the use of conditional knockout techniques, 3) various non-equivalent sources of ECs are used for in vitro experiments, and 4) quite paradoxically very few ECs express FGF receptors in vivo, with expression restricted mainly to large vessels and within less than 20% of the cells (however ex vivo cultured ECs do express high levels of FGFR1)[65, 66]. Thus, FGFR1 expression by ECs has been largely assumed to be restricted to proliferating cells but the physiological significance and causality between proliferation and receptor expression remains debatable and may reflect an indirect effect.

The first evidence that the mitogenic effect of FGF-2 on ECs may be indirect was provided when it was shown that FGF-2 upregulated VEGF expression and blocking of VEGF using antibodies completely abolished FGF-2-induced ECs proliferation in vitro and angiogenesis in vivo [67]. Using embryoid bodies derived from FGFR1^−/−^ ES cells, which under appropriate conditions recapitulate haemangioblasts differentiation in vitro, Magnusson et al. (2005,2007) demonstrated that FGFR1 was indeed not necessary for ECs differentiation and vascular plexus formation, it was however required for hematopoietic development [68, 69]. Moreover, the FGFR^−/−^ embryoid bodies contained more blood vessels and ECs derived from them proliferated faster. In a more recent study, Murakami et al. (2008) used soluble FGFRs in vivo to demonstrate the importance of FGF signaling in maintaining vascular integrity, more specifically in maintaining adherens and tight junctions between ECs [70]. However because FGFRs are poorly expressed by ECs in vivo, it is not clear whether blocking FGF signaling affected ECs directly or rather the underlying pericytes and vascular smooth muscle cells. The same group recently demonstrated that one of the effects of FGF stimulation on ECs was actually to modulate their responsiveness to VEGF, in part by upregulation of VEGFR2 [71]. These and other studies (reviewed in [72]) indicate that one of the major roles of FGF signaling in ECs might be to orchestrate a complex crosstalk between ECs and pericytes by not only modulating the production of other growth factors (amongst which PDGF and VEGF appear pivotal) but also the responsiveness of the cells to these factors. Quite interestingly, FGF also appears to inhibit senescence in ECs. Indeed, senescent HUVECs loose responsiveness to FGF stimulation [73] whereas primary ECs and HUVECs upregulate telomerase in response to FGF-2 but not VEGF [74]. Moreover, HUVECs cultured without growth factors of with VEGF alone have been shown to enter senescence within 15 population doublings whereas the single addition of FGF-2 allowed the cells to proliferate up to 40 population doublings before onset of senescence [75].

From what has just been described and contrary to widespread belief, it is obvious that FGFs are not mere mitogens for ECs and in fact they may even exert their effects mostly by indirect means, whether by acting on accessory cells such as pericytes or by modulating the activity of other growth factors. This is supported by the low FGFR1 expression by ECs in vivo and the fact that FGFR^−/−^ embryoid bodies show no obvious defects in angiogenesis. It could be that FGF signaling serves to protect ECs from cellular senescence during active proliferation or that FGFR1 is only expressed in endothelial progenitors, but this requires more investigations. A fundamental requirement that needs to be addressed before answering some of these unresolved questions is a better understanding of the heterogeneity of ECs in vivo and in vitro, of their phenotypic differences and various physiological roles. As not all ECs are the equivalent, this would enable to test the effects of FGF stimulation on specific subsets of ECs.

### Hematopoietic stem cells and blood cells

As already mentioned, hematopoietic stem cells (HSCs) arise from haemangioblasts located in the AGM and yolk sac during embryonic development, before undergoing a journey that will take them to the placenta and fetal liver and eventually the bone marrow shortly before birth [64]. In this latter location, they will self-renew and give rise to the billions of new blood cells required per day, for the lifespan of the organism. Although the importance of FGF signaling in hematopoiesis as long been recognized and studied, its specific role in HSC self-renewal, proliferation and lineage commitment remains controversial to this day [76, 77].

FGF-2 was initially shown to be a mitogen for multipotent progenitors from bone marrow, mostly in the myeloid lineage [78-80]. Although ineffective by itself, it was thought to potentiate the effects of other growth factors and thus act as a permissive factor. Indeed, it appears to synergize with IL3, GM-CSF and EPO to increase the production of CFU-GEMM and with SCF and GM-CSF to stimulate myelopoiesis [81]. FGFR1 and 2 were also found on most blood cells, including megakaryocytes, platelets, macrophages, granulocytes and to a lesser extent on B and T lymphocytes. The indirect effect of FGF stimulation on blood cell proliferation was supported by the fact that FGF-1 and 2 stimulated the proliferation of megakaryocytes and erythroleukemia cells but this effect was blocked by anti-IL6 antibodies [82]. The stimulatory effect of FGF on megakaryopoiesis nevertheless appears very potent since daily injections of recombinant FGF-4 or FGF-4 adenovirus completely rescues thrompocytopenia in TPO deficient mice [83]. In this model, FGF-4 increased megakaryocytes adhesion to blood vessels and their subsequent maturation.

While the mitogenic effect of FGF on myeloid cells is obvious, its effect on embryonic and adult HSCs is more controversial. Berardi and colleagues(1995)[84] found no stimulatory effect of FGF on human CD34+ cells from bone marrow, whereas Wagner et al. (2011)[85] observed a greater proliferation and NOD-SCID reconstitution of CD34+ cell derived from human cord blood when expanded on mesenchymal stem cells with TPO, SCF and FGF1. Obviously, in the latter study we cannot exclude an indirect effect of FGF through the feeder layer as FGFRs expression in the two cell types was not tested. More recently, it was shown that a combination of SCF, TPO, FGF1, IGFBP2 and Angptl5 was necessary to expand serially transplantable CD34+CD133+ cells from cord blood [86, 87].

In mice, it was first shown that FGF-2 signaling through FGFR1 was required for hematopoietic commitment of haemangioblasts derived from ESCs [69, 88]. Moreover, Miller et al. (2003) showed that all long-term repopulating cells were found in the c-kit+/Sca-1+/Lin- (KSL) FGFR1+ fraction of bone marrow cells, although only 0.2% of these were of hematopoietic origin [89]. These cells expressed FGFR1, 3 and 4 and could be expanded for 4 weeks in vitro in the presence of FGF1 while maintaining their multipotentiality in vitro and in vivo, although this was not tested in single-cell transplantations. In another study, a constitutively active FGFR2 was expressed in hematopoietic cells under the Tie2 promoter. These mice showed no obvious hematopoietic defects but their KSL cells possessed increased multilineage reconstitution and decreased apoptosis after transplantation into wild-type animals [90]. On the other hand, FGF-4 and 8 were found to inhibit blood formation in chick embryos whereas inhibition of FGF signaling induced ectopic blood island formation [91]. Furthermore, FGF stimulation has been shown to suppress the expansion of activated HOXB4-overexpressing HSCs derived for ESCs or adult marrow [92]. In this same study however, FGF was found to stimulate the proliferation of normal HSCs not overexpressing HOXB4.

The wide variety of cell types used, purity of cell populations, culture conditions and endpoint assays to determine the stemness of HSCs in the studies described here probably explain the controversy regarding the role of FGF in hematopoiesis. As our definition of HSCs constantly evolves and better techniques are available to study these cells at near purity [93], it will clearly be necessary to revisit the role of FGF signaling in better defined populations using gold standard assays such as single-cell assays and transplants. Despite apparently contradictory results however, it is probably safe to say that as in other tissues, FGF could have stage-specific effects, with stimulation of self-renewal in stem cells and early progenitors and pro-differentiation effects on later progenitors. Although to our knowledge there has been no published studies linking FGF signaling and senescence of HSCs, it is intriguing that MDM2 (which we have found to mediate FGF-induced inhibition of senescence in MSCs)[58] was found to be required for HSCs survival following their colonization of bone marrow [94]. Indeed, MDM2 knockout mice expressing a hypomorphic p53 allele to rescue their embryonic lethal phenotype die shortly after birth from marrow failure, showing extensive medullary senescence and hypocellularity. Since MDM2 is a negative regulator of p53 and may thus improve HSCs self-renewal [95], and because senescence leads to decreased HSC function with aging [96, 97], it would be interesting and potentially of therapeutic use to see if FGF signaling also directly modulates MDM2 activity in HSCs.

## FGF SIGNALING IN ECTODERMAL STEM CELLS AND TISSUES

The importance of FGF signaling in central and peripheral nervous system both during development and postnatal life has been long recognized. In addition to the difficulties in studying FGF signaling mentioned above for other tissues, our knowledge of brain development and neural stem cells has greatly evolved in the last two decades rendering previous conclusions obsolete or in any case requiring re-evaluation. The skin is another ectoderm-derived tissue containing various stem cell populations where FGFs and their receptors are widely distributed, yet very little is known about the precise role of FGF signaling in skin homeostasis or repair and it will not be discussed here. This section will review what is known about FGF signaling in neural tissue.

### Neural stem cells and the nervous system

During embryonic development, the nervous system arises shortly after gastrulation from neuroepithelium located along the dorsal midline of the embryo (the prospective neural plate) and then folds into the neural tube before undergoing various patterning events and specification. Whereas most neural cells in early embryonic development are multipotent (they can give rise to both neurons and glia), neural stem cells (NSCs) become restricted to specific areas later in development and postnatal life: the cerebellum, the subgranular zone (SGZ) of the dentate gyrus in the hippocampus, and subependymal zone (SEZ, subventricular zone [SVZ] during development) lining the lateral ventricles [98, 99]. NSCs in the cerebellum are only present for a few weeks in postnatal animals whereas NSCs in the SGZ produce excitatory granule neurons for their entire lifespan. NSCs in the SVZ are thought to give rise to most central nervous system neurons and glia in the developing mouse telencephalon and continue lifelong to provide neural progenitors that migrate along the rostral migratory stream to the olfactory bulb, a major zone of adult neurogenesis [100]. These cells have a radial glia identity during development and throughout neurogenesis, after which they adopt an astroglial stem cell (the adult NSCs in the SEZ) or ependymal phenotype. This glial identity of NSCs is significant since it implies that other adult glia such as NG2 glia or even astrocytes, may under certain circumstances de-differentiate to a more primitive multipotent state to participate in tissue repair, although this remains to be proven in vivo [101].

FGF signaling has long been acknowledged for its neural induction role in the developing embryo [102, 103] as well as for its mitogenic/self-renewal effect on NSCs in vitro and in vivo [104-106]. Indeed, FGF-2 in combination with EGF is ubiquitously used to expand NSCs in the neurosphere assay. At least 10 of the 23 FGF ligands have been described to be expressed in the brain. FGFR1 is expressed as early as E8.5-9.5 in mouse telencephalon and persists in the ventricular zone and dentate gyrus later on [107, 108]. Expression of FGFR2 and 3 have also been reported and seem to be highly expressed by glial cells, mostly in the SEZ and SGZ but also around brain lesions following trauma [105, 109]. The expression of FGFRs and their ligands appears very dynamic and may have stage specific effects during development and adult life [110-112]. Interestingly, FGF-2 and HSPGs have been found closely associated with proliferating NSCs in vivo and may also regulate NSCs self-renewal in vitro [106, 113]. Moreover, radial glia in zebrafish appear to have increased FGF signaling [114].

Aging is usually associated with a decline in cognitive functions including memory as well as decreased regenerative capacity. Aging has also been associated with decreased NSCs or progenitors number and self-renewal capacity in the SEZ (see [115] and references therein), which may correspond to increased NSC senescence in vivo [116, 117]. The number of FGFR2+ glial cells is also decreased in the olfactory bulb, SEZ, cerebellum and hippocampus of aged mice [109]. Interestingly, administration of FGF-2 either intraventricular or subcutaneous appears to increase neurogenesis and NSCs proliferation in the SEZ and SGZ of both young and aged mice [118, 119]. Furthermore, FGF has been shown to protect against memory impairment in senescence-accelerated mice, a murine model of aging [120, 121]. It might be relevant to point out that at least one strain of senescent-accelerated mice with an increased neurological senescent phenotype resembling human aging has been shown to have a 50% deletion of its FGF-1 gene, leading to the complete absence of the protein in the brain.

From the studies presented here, it is obvious that FGF signaling plays a major role in regulating NSCs proliferation and self-renewal in vitro and in vivo. There is also ample evidence that it may have a pro-differentiation effect on more committed progenitors. As our understanding of NSCs and brain development increases, it will be possible to specify these roles more precisely by using conditional knockout techniques. The strong association between FGF signaling and NSCs senescence and aging should serve as an incentive for these future studies in the hope of developing new treatments against neurological degeneration and possibly brain repair after trauma.

## CONCLUDING REMARKS

Throughout this review, we have seen that FGFs and their receptors play important roles in the embryonic development, homeostasis and repair of most organs. The effects of FGF signaling can be in part attributed to the stimulation of self-renewal in endogenous somatic stem cells within these organs, but there is also much evidence that FGF signaling also plays a role in the concomitant inhibition of cellular senescence in stem cells. The evidence presented here also suggests a role of FGF signaling in the more committed cells downstream of stem cells, a role that appears to stimulate differentiation. Moreover, in most cell types studied, FGF seems to play a permissive role rather than a direct inductive or instructional role, usually by modifying the responsiveness of the cells to other factors or by potentiating and synergizing with other signals. That seems to hold true in both stem cell self-renewal and differentiation of more committed cells. Although not discussed here FGF signaling also plays a major role in endodermal tissues, in lung patterning, liver and pancreas specification, and in self-renewal of stem cells in the intestinal crypts for instance. However, these tissues have not received as much attention in publications and little is yet known about the roles of FGF signaling in their maintenance into adulthood. Nevertheless, the roles we have described for FGF signaling in regulation of stem cells self-renewal and aging, the fact that our definitions and understanding of these same stem cells is better refined every day, and the development of more advanced reagents and techniques to study stem cells should stimulate more research into this field.

The roles played by FGFs and FGFRs in aging or age-related disorders are gradually being unveiled. This is exemplified by the accelerated aging-like phenotype of FGF-23 knockout mice [122] and by the decreased expression of FGF ligands and receptors (or at least blunted responsiveness to FGF signaling) in aged tissues such as brain, bone and skin [123-127]. Since FGF signaling is so potent at inducing stem cell self-renewal and inhibiting their senescence, therapeutic targeting of FGF signaling components by recombinant proteins, gene therapy or small molecules could well be used to reverse some of the effects of aging. In fact, various FGFs are currently being tested therapeutically for a number of age-related disorders such as cardiovascular diseases, diabetes, osteoarthritis, chronic kidney disease, Parkinson's disease and mood disorders (reviewed in [128], also see http://clinicaltrials.gov). Most of the stem cell populations described in this review have enormous therapeutic potential and increasing our capacity to harness their power to address unmet medical needs and reduce human suffering is the holy grail of current biomedical research. FGFs could well be added to the toolbox required to achieve this goal.
